# Skin Lipid–Microbe Interplay Links *Staphylococcus hominis* to Barrier Control in Adult Atopic Dermatitis

**DOI:** 10.1111/all.70028

**Published:** 2025-08-28

**Authors:** Madhumita Bhattacharyya, Felix Lauffer, Manja Jargosch, Kristina Frey, Mahsa Shahidi Dadras, Theresa Raunegger, Sophia Wasserer, Carsten B. Schmidt‐Weber, Tilo Biedermann, Kilian Eyerich, Stefanie Eyerich, Claudia Traidl‐Hoffmann, Christian Klose, Matthias Reiger, Natalie Garzorz‐Stark

**Affiliations:** ^1^ Environmental Medicine – Institute of Environmental Medicine and Integrative Health, Faculty of Medicine University of Augsburg and University Hospital of Augsburg Augsburg Germany; ^2^ Institute of Environmental Medicine Helmholtz Center Munich, German Research Center for Environmental Health Augsburg Germany; ^3^ Department of Dermatology and Allergy LMU Hospital Munich Munich Germany; ^4^ Department of Dermatology and Allergy Technical University of Munich Munich Germany; ^5^ ZAUM – Center of Allergy and Environment Technical University of Munich and Helmholtz Center Munich, Member of the German Center for Lung Research (DZL) Munich Germany; ^6^ Department of Dermatology and Venerology, Medical Center—University of Freiburg, Faculty of Medicine University of Freiburg Freiburg Germany; ^7^ Division of Dermatology and Venereology, Department of Medicine Solna, and Center for Molecular Medicine Karolinska Institutet Stockholm Sweden; ^8^ Christine Kühne–Center for Allergy Research and Education (CK‐CARE) Davos Switzerland; ^9^ Lipotype GmbH Dresden Germany

**Keywords:** atopic dermatitis, lipidomics, microbiome, *Staphylococcus hominis*

## Abstract

**Background:**

Skin surface lipids and commensal microbes are essential for the epidermal barrier, but their mutual interactions remain poorly understood.

**Methods:**

We conducted high‐resolution shotgun lipidomics of tape strips from lesional and non‐lesional atopic dermatitis (AD) skin and healthy controls. Lipidomic data were integrated with 16S amplicon sequencing to construct lipid–microbe interaction networks.

**Results:**

AD skin showed disease‐specific lipid–microbe correlations, with less diverse interactions in lesional compared to non‐lesional and healthy skin. *Staphylococcus hominis* ( *S. hominis* ) negatively correlated with non‐hydroxy—dehydrosphingosine (NdS) 18:0;2/24:0;0 and positively with diacylglycerol (DAG) 18:1;0_18:1;0 and DAG 16:0;0_18:1;0. In vitro co‐cultures of reconstructed human epidermis (RHE) with AD skin‐derived T cell supernatant (TCS) and *S. hominis* reduced RHE thickness, spongiosis, and NdS 18:0;2/24:0;0 levels. Furthermore, *S. hominis* directly lowered NdS 18:0;2/24:0;0 levels in lesional AD skin tape samples, and reversed type 2 inflammation and lipid metabolism gene expression in TCS‐stimulated RHE.

**Conclusions:**

These findings identify *S. hominis* as a key regulator of lipid–microbe interactions in AD, influencing epidermal inflammation and differentiation.

## Graphical abstract

This study investigates mutual interaction of skin microbes and surface lipids in AD using the advanced method of shotgun lipidomics with ultra‐broad coverage of lipids and 16S amplicon sequencing of tape strips taken from lesional and non‐lesional AD and healthy controls. Multi‐omics integration of microbiome and lipidome from lesional and non‐lesional AD as well as healthy skin reveals disease‐specific interaction networks. *S. hominis* correlates with key lipid species and functional validation in RHE and bacterial culture models confirms *S. hominis* as a regulator of skin lipid homeostasis. Abbreviations: AD, atopic dermatitis; DAG, diacylglycerol; LS, lesional; NdS, non‐hydroxy–dehydrosphingosine; NL, non‐lesional; RHE, reconstructed human epidermis; RNAseq, RNA sequencing; *S. hominis*, *Staphylococcus hominis*; TCS, T cell supernatant; US, unstimulated.

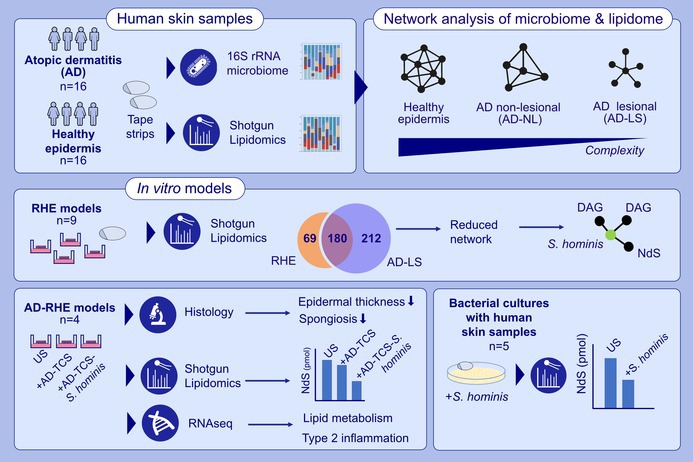

**Abbreviation**
AD: atopic dermatitis

## 1 Introduction

An intact skin barrier is a prerequisite to protect the human body from water loss and exogenous noxae such as allergens and pathogens [1]. Terminally differentiated keratinocytes interconnected via protein‐crosslinking, lipid‐containing extracellular matrix, and microbial communities on the skin surface are integral constituents of the skin barrier [2]. Disbalances in this finely tuned system, which shows strong topographic variations, contribute to common skin diseases such as atopic dermatitis (AD) [3]. Lipid groups and microbial species have been studied separately from each other in disease and health, revealing differences in composition and richness of lipids and microbes. It has been shown, for example, that the dry skin of AD was characterized by an increase of short‐chain free fatty acids (FFA), short‐chain non‐hydroxy‐sphingosine (NS) ceramides, and sphingomyelins, whereas the proportion of corresponding long‐chain species of sphingolipids and FFA decreased compared to healthy controls [4]. With regards to the skin microbiome, pioneering publications revealed that the diversity of microbial communities was reduced in favor of *S. aureus* , which was detected at AD predilection sites, in particular during disease flares [5]. However, to better understand the interplay between lipids and microbes and their contribution to disease, both components need to be investigated comprehensively and together in individuals. Following this approach, Baurecht et al. [3] found that, in AD skin, levels of long‐chain unsaturated FFA strongly correlated with Propionibacteria and Corynebacteria abundance. Here, we set forth to study the mutual interaction of skin microbes and surface lipids in AD using the advanced method of shotgun lipidomics with ultra‐broad coverage of lipids and 16S amplicon sequencing of tape strips taken from lesional and non‐lesional AD and healthy controls. By this, we created so far unknown correlation networks of lipids and microbes in AD. Moreover, using co‐cultures of bacterial species and reconstructed human epidermis (RHE), the interplay of *Staphylococcus hominis* , skin barrier, and the epidermal lipid non‐hydroxy‐dehydrosphingosine NdS 18:0;2/24:0;0 was exemplarily validated in vitro, indicating that lipid–microbe‐specific interactions may contribute to disease pathology.

## 2 Materials and Methods

### 2.1 Study Design and Sampling

This pilot study investigated the lipid and microbiome signatures of AD. Sixteen AD patients (mean age 44.8 ± 16.3 years, range 19–72) and 16 age‐ and sex‐matched healthy controls (HE) were included (Table S1). AD diagnosis was confirmed at least 6 months prior, with exclusion criteria including systemic AD therapy and topical treatments within 2 weeks prior to sampling. Emollients were avoided 24 h before sampling. All participants provided written consent, and the study was approved by the ethics committee of the School of Medicine and Health, Technical University of Munich (154/16S).

Skin samples were collected using CUDERM D‐Squame discs (tapes). After site cleaning with a D100 disc, three D101 discs were applied to lesional (LS) and non‐lesional (NL) skin of each AD patient. Samples were primarily taken from the hand or leg, with some from the chest or back. LS and NL samples were collected from comparable locations within patients, and HE samples matched LS sites. The tapes were stored at −20°C until further processing.

### 2.2 Lipid Extraction and Analysis

Extraction, quantification, and identification of lipid molecules was performed by Lipotype GmbH according to the protocol based on the shotgun lipidomics technique published by Sadowski et al. [6]. In brief, samples were spiked with lipid internal standards prior to methanol‐based extraction. After drying and re‐suspending in the Mass spectrometry (MS) acquisition mixture, lipid extracts were subjected to MS analysis. Mass spectra were acquired on a hybrid quadrupole/Orbitrap mass spectrometer equipped with an automated nano‐flow electrospray ion source in both positive and negative ion modes.

### 2.3 Lipid Data Processing

Lipid identification via LipotypeXplorer used raw mass spectra, analyzing intact masses (MS‐only) and fragment masses (MSMS) to determine lipid subspecies. Lipids underwent filtering based on mass accuracy, detection threshold (≥ 50% per group), signal‐to‐noise ratio (≥ 5‐fold), and background levels (≥ 5‐fold signal to blank).

Molecular lipids were annotated according to their molecular composition as NAME<sum of the carbon atoms in the hydrocarbon moiety>:<sum of the double bonds in the hydrocarbon moiety>;<sum of hydroxyl groups>, and quantified using class‐specific internal standards. Lipid species amounts (pmol) were summed per class. Lipids were categorized into 3 groups, 7 classes, and 12 subclasses (Figure S1). The final dataset included 513 lipid molecules (median per sample: 2.613 pmol, range: 0.0125–4880 pmol).

### 2.4 DNA Extraction and 16S rRNA Sequencing for Microbiome

For microbiome analysis, collected tapes (D‐Squame standard sampling disc D100 diameter 1 cm) were stored in 500 μL Stool DNA Stabilizer Solution (Stratec) at −80°C and analyzed using 16S (variable region V1–V3, primers 27F‐YM and 534R) amplicon‐based next‐generation sequencing. For DNA extraction, the QIAamp UCP Pathogen Kit (Qiagen) was used. Lysis was performed using Precellys Evolution (Bertin Technologies: Montigny‐le‐Bretonneux, France).

### 2.5 DNA Sequencing Data Analysis for Microbiome

16S rRNA sequencing was performed on the Illumina MiSeq platform (2 × 300 bp reads, MiSeq Reagent Kit v3 600 cycles). Data were processed using the DADA2 (version 1.16.0) pipeline in R (version 3.6.4), including filtering, trimming, denoising, and merging of paired‐end reads. Sequences < 200 bp and chimeras were removed.

Amplicon sequence variants (ASVs) were annotated using AnnotIEM, comparing alignments against EzBioCloud, NCBI, RDP, and Silva. Singletons, contaminants, and eukaryotic ASVs were excluded for additional quality control [7]. The final dataset contained 434,042 reads (median: 8192.5, range: 5213–19,346) assigned to 613 ASVs (median: 62, range: 29–120).

For consistency with lipid analysis, only ASVs present in ≥ 50% of any subgroup (LS, NL, HE) were included. Abundances were summed at the species level, yielding 86.6% of total reads (median: 7000.5, range: 4209–18,979) across 49 ASVs (median: 36, range: 22–48), distributed among 19 families. Of these, 42 were classified to species level, and seven to genus level. Absolute quantification of the 16S rRNA gene was carried out as described previously [7].

### 2.6 *Staphylococcus hominis* Culture and Skin Tape Sample Incubation With *S. hominis*

Skin samples were collected from neighboring areas of the same lesional site in patients with AD using CUDERM D‐Squame discs D101. *Staphylococcus hominis* (DSM 20328, subsp. hominis, type strain; ATCC 27844) was grown in Lysogeny Broth (LB) [8] at 37°C. Medium was solidified by adding 1.5% (w/v) agar. Inocula from frozen glycerol cultures in LB medium were grown overnight at 37°C to log‐phase growth. Bacterial cell number was calculated from the absorbance of the suspension at 600 nm (OD600 = 1 equals 6 × 10^7^ cells/mL). Overnight cultures of the strain were diluted to an OD at 600 nm of 0.1 in LB medium, and 100 μL of a 1:500 times dilution was plated onto separate LB agar plates (90 mm in diameter) yielding approximately 200–2000 colonies per plate. After a 2‐h initial incubation of bacteria, D‐Squame discs were placed faced‐down onto the microbial lawns and incubated for another 6 h. As controls, one disc was incubated on sterile LB agar (Ctrl‐Agar), and another one was left unexposed (Ctrl). All discs were stored at −20°C until further processing.

### 2.7 RNA Sequencing, Rescue Score and Pathway Analyses

RNA from skin models (RHE) was isolated using the miRNeasy Mini Kit (Qiagen) for cell culture cells according to the manufacturer's protocol. Library preparation, including poly(A) enrichment and rRNA depletion, was performed at BGI (Shenzhen/Global) using their proprietary DNBSEQ NGS workflow. Sequencing was carried out in paired‐end mode (2 × 100 bp), with an average depth of ≥ 30 million reads per sample. Reads were aligned to the human reference genome (GRCh38/hg38) using the HISAT2 aligner. The average genome alignment rate across samples was 98.67%, with 89.24% of reads mapping to annotated genes. A total of 16,588 genes were detected in the dataset. RNAseq count data were normalized with edgeR's TMM method and transformed to log_2_CPM. Low‐expression genes (CPM ≤ 1 in > 50% of samples) were excluded, retaining those with CPM > 1 in ≥ 3 samples. Differential expression analysis was performed using the Limma‐voom pipeline with a design matrix accounting for donor effects. Voom was used to estimate precision weights, followed by linear modeling (lmFit), contrast definition, and moderated *t*‐statistics and Benjamini–Hochberg adjusted *p*‐values via eBayes. Intra‐donor correlation was modeled using duplicateCorrelation and incorporated in a second linear model fit. To quantify the extent to which *S. hominis* co‐culture “rescued” TCS‐induced transcriptional changes, we computed a rescue score for each gene:

$$\text{Rescue score}=|\log_{2}\text{FCTCS}\;\mathrm{vs}.\mathrm{US}|-|\log_{2}\text{FCTCS}+S.\mathrm{hom}\;\mathrm{vs}.\mathrm{US}|$$

Genes with rescue score > 0 and adjusted *p* < 0.05 (TCS vs. unstimulated control) were flagged as rescued. Based on expression patterns, genes were classified as: (1) Rescued genes: TCS‐induced changes reversed by *S. hominis* and (2) Non‐rescued genes: TCS‐induced changes enhanced by *S. hominis* . Pathway enrichment analysis was performed separately for both groups using ConsensusPathDB (Wikipathways, Reactome, PID; *p* < 0.01; min. overlap = 2) [9]. Networks were built from the top 20 enriched pathways. Representative genes were visualized using boxplots.

### 2.8 Correlation Network Analysis

Correlation analysis was conducted at four levels: (i) lipid classes vs. microbe families, (ii) lipid classes vs. microbe species, (iii) lipid species vs. microbe species, and (iv) lipid species vs. microbe families. Networks were generated from Pearson's correlation matrices (PCC ≥ 0.7 or ≤ −0.7) across all samples per group using in‐house programs (Threshold for inclusion—0.7 ≥ PCC ≥ 0.7). Visualization and mapping were performed with Cytoscape v3.8.0.

Centrality analysis (CentiScaPe 2.2) assessed degree (number of connections), betweenness (shortest paths through a node), closeness (node proximity), and radiality (network modularity). Higher closeness indicates tightly interconnected nodes, while higher radiality suggests a more modular network with less interconnected nodes. Diameter and shortest path were used to assess network size and density, with higher diameter indicating larger network size and shorter shortest path indicating dense connectivity.

### 2.9 Statistical Analysis

Statistical analyses were conducted using R (v4.0.3), MicrobiomeAnalyst, and GraphPad V8. Microbiome analyses were performed at the species level, while lipidome analyses included both molecular lipid and lipid class levels. Total sum scaling (TSS) was used to normalize lipid (pmol) and microbiome (read count) data.

For comparisons of unpaired data across more than two groups (e.g., microbiome and lipid class abundance across LS, NL, and HE), the Kruskal–Wallis test (two‐sided, *p* ≤ 0.01) was applied, followed by post hoc Dunn's test where applicable. Mann–Whitney *U* tests were used for pairwise comparisons between unpaired groups (e.g., LS vs. HE, NL vs. HE in network centrality analyses). In vitro data was presented as mean ± SEM and compared as indicated in figure legends. For in vitro experiments using RHE models, paired data from *n* = 4 independent donors were analyzed using paired two‐tailed *t*‐tests. For normally distributed unpaired data (e.g., gene expression in independent RHE models), one‐way ANOVA was used. Significance thresholds: *p* < 0.05 (*), < 0.01 (**), < 0.001 (***), < 0.0001 (****).

Beta diversity was assessed via Bray–Curtis dissimilarities and visualized using PCoA, with significance tested via PERMANOVA (1000 permutations). Microbial taxonomic distributions were shown as stacked bar plots, summarizing the top ten most abundant families/species, with others grouped as “Others.” Lipid distributions were plotted as relative abundance (pmol) of 16 lipid classes. Variance estimation was performed using the Bioconductor package Variance Partition.

## 3 Results

### 3.1 Lesional Skin of AD Is Characterized by a Distinct Lipid Profile

**FIGURE 1:**
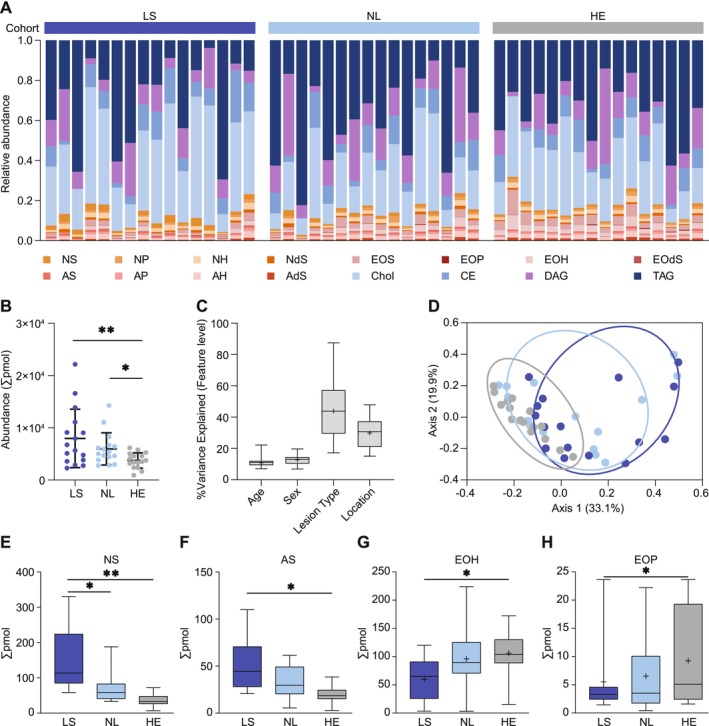
Lipid profiles of lesional atopic dermatitis (LS), non‐lesional AD (NL) and matched healthy controls (HE). Lipid composition is plotted as frequency (in percentage) on the class level (A) and total abundance is shown as scatterplots (B). Healthy control samples in each cohort were listed in the same order as corresponding matching lesional/non‐lesional samples. Variance analysis showing the contribution of the factors age, sex, lesion type, and sampling site to the variance within the lipid dataset (C). Beta diversity analysis shows differences in lipid profiles of LS, NL, and HE (D). Four differentially abundant lipid classes (NS, AS, EOH and EOP) are presented as box‐whisker plots (E–H). AdS, alpha‐hydroxy‐dehydrosphingosine; AH, alpha‐hydroxy‐6‐hydroxysphingosine; AP, alpha‐hydroxy‐phytosphingosine; AS, alpha‐hydroxy‐sphingosine; CE, cholesteryl esters; Chol, cholesterol; DAG, diacylglycerol; EOdS, omegahydroxy‐dehydrosphingosine; EOH, omegahydroxy‐6‐hydroxy‐sphingosine; EOP, omegahydroxy‐phytosphingosine; EOS, omegahydroxy‐sphingosine; NdS, non‐hydroxy‐dehydrosphingosine; NH, non‐hydroxy‐6‐hydroxysphingosine; NP, non‐hydroxy‐phytosphingosine; NS, non‐hydroxy‐sphingosine; TAG, triacylglycerol; for all pairwise comparisons, a non‐parametric Kruskal–Wallis test was performed, and a *p*‐value < 0.05 is indicated by *; a *p*‐value < 0.01 is indicated by **.

**FIGURE 2:**
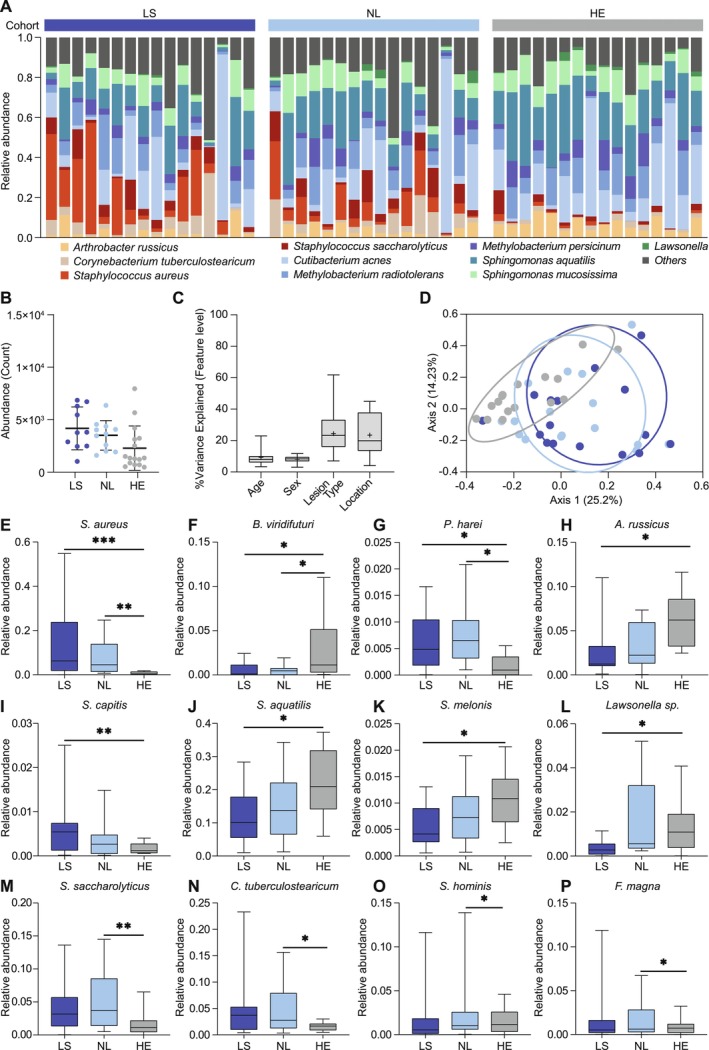
Microbial profiles of lesional atopic dermatitis (LS), non‐lesional AD (NL) and matched healthy controls (HE). Microbe composition is plotted as frequency (in percentage) on the species level for the top 10 most abundant species (A) and total abundance as derived by qPCR is shown as scatterplots (B). Healthy control samples in each cohort were listed in the same order as corresponding matching lesional/non‐lesional samples. Variance analysis showing the contribution of the factors age, sex, lesion type, and sampling site to the variance within the full microbiome dataset (C). Beta diversity analysis shows differences in microbial profiles of lesional and non‐lesional skin as well as of healthy controls (D). Twelve differentially abundant bacterial species are presented as box‐whisker plots (E–P). For all pairwise comparisons, a non‐parametric Kruskal–Wallis test was performed, and *p*‐value < 0.05 is indicated by *; *p*‐value < 0.01 is indicated by **; *p*‐value < 0.001 is indicated by ***.

To test the hypothesis that epidermal lipids and skin commensals are interconnected, we first analyzed skin lipid (Figure 1) and microbiome profiles (Figure 2) using non‐invasive tape‐stripping in a study cohort of 16 AD patients and 16 age‐ and sex‐matched healthy controls (Table 1). Lesional AD (LS) and nonlesional AD (NL) samples were clearly dominated by lipids of the cholesterol class (Chol) (Figure 1A) and lipid abundance in LS samples was significantly higher than in NL and matching healthy samples (HE) (LS: mean abundance per sample = 7968, HE, mean abundance = 3753 pmol, *p* ≤ 0.001, NL, mean abundance = 5964 pmol, *p* ≤ 0.01) (Figure 1B). Hierarchical clustering on lipid abundance showed abundance of cholesterols, non‐hydroxyceramide subspecies non‐hydroxy‐sphingosines (NS), non‐hydroxy‐dehydrosphingosines (NdS), and non‐hydroxy‐6‐hydroxyshingosines (NH) as well as alpha‐hydroxyceramide subclass alpha‐hydroxy‐sphingosine (AS) in AD samples, with a decreasing tendency of abundance towards NL and HE (Figure S2A). Omega‐hydroxyceramide subclasses omegahydroxy‐6‐hydroxy‐sphingosine (EOH), omegahydroxy‐sphingosine (EOS), non‐hydroxyceramide subspecies non‐hydroxy‐phytosphingosine (NP) and alpha‐hydroxyceramides of the alpha‐hydroxy‐dehydrosphingosine (AdS), alpha‐hydroxy‐6‐hydroxysphingosine (AH), and alpha‐hydroxy‐phytosphingosine (AP) subclass showed an increasing trend of absolute abundance from LS to NL and HE (Figure S2A). Overall, the diversity of lipids per sample, as measured by alpha diversity indices Richness, Shannon Index, and Simpson's Index, was lowest in LS, with an increasing tendency towards NL and HE (Figure S2B–D).

**TABLE 1:** Characteristics of microbe‐lipid correlation networks in AV.

Groups		AD		HE	Sum
		LS	NL		
Sample size	*N*	16	16	16	48
Age, years	*N*	16	16	16	48
	Minimum	19	19	20	—
	Maximum	72	72	80	—
	Mean	44.81	44.81	45.56	—
	Median	48	48	47	—
	SD	16.34	16.34	17.48	—
Sex, *N* (%)	*N*	16	16	16	48
	Male	7 (43%)	7 (43%)	7 (43%)	21 (43%)
	Female	9 (56%)	9 (56%)	9 (56%)	27 (57%)
Sampling site, *N* (%)	Hand	8 (50%)	9 (56%)	8 (50%)	25 (52%)
	Leg	4 (25%)	4 (25%)	4 (25%)	12 (25%)
	Abdomen	1 (6%)	1 (6%)	1 (6%)	3 (6%)
	Back	3 (18%)	2 (12%)	3 (18%)	16 (33%)

Lipid and microbial profiles may not only depend on disease status but also on sampling site, age, and sex. In our study, age and sex accounted for 9% and 8.4% of the variance in the lipidome, respectively, while sampling site contributed 29.4% and lesion type 38.2% (Figure 1C). Within the AD cohort, beta diversity analysis (PCoA) showed a significant difference in lipid composition of HE as compared to matching LS (*p* = 0.00032) and NL (*p* = 0.0053), but also between LS and NL (*p* = 0.062) (Figure 1D). Signature lipids in AD included AS (*p* = 0.0105) and NS (*p* = 0.0061) (Figure 1E,F) with specific enrichment of NS 18:1;2, NdS 18:0;2/16:0;0, DAG 18:0;0_18:2;0, DAG 18:1;0_18:2;0, NH 17:1;3/18:0;0, and NH 19:1;3/15:0;0 (Figure S3A–L). EOH showed lowest levels in LS as compared to NL and HE (*p* = 0.006). In addition, EOP was also found lowest in LS as compared to NL and HE (*p* = 0.006) (Figure 1G,H).

### 3.2 Microbial Composition of Lesional AD Skin as Compared to Non‐Lesional and Healthy Skin

Next, we analyzed skin microbiome signatures from the same sites as lipid samples. LS and NL showed increased microbial abundance, particularly *Staphylococcal* species, compared to HE, while *Sphingomonas* increased from LS to NL and HE (Figure 2A,B, Figure S2E). Unlike the lipidome, alpha diversity (richness) was highest in LS as compared to NL and HE (Figure S2F–H). Age and sex contributed minimally, while sampling site (24.36%) and lesion type (24.36%) accounted for most microbiome variance (Figure 2C). Beta diversity analysis showed significant microbiome differences between all groups (*p* < 0.01), with LS vs. HE (*p* = 0.0071) and NL vs. HE (*p* = 0.0033) being significant (Figure 2D). Twelve signature microbes were identified (Figure 2E–P). *S. aureus* was enriched in LS, while *S. melonis* , *A. russicus*, and *S. aquatilis* were underrepresented. Interestingly, *S. hominis* and *F. magna* were more abundant in NL than in HE (*p* = 0.019). To better understand the contribution of *Staphylococcus* species beyond *S. aureus* in AD, we grouped species based on published evidence of their pro‐inflammatory or protective properties. Inflammatory species ( *S. aureus* [10], *S. caprae* [11], *S. petrasii* [12]) were compared to commensals with proposed protective functions and those which are found on healthy skin and currently lack evidence of proinflammatory activity ( *S. warneri* [13], *S. equorum* [14], *S. capitis* [13], *S. hominis* [15], *S. epidermidis* , *S. saccharolyticus* [16]) and their relative abundances were analyzed across LS, NL, and HE skin. The ratio of inflammatory to protective species was significantly elevated in LS than in NL and HE (Figure S4A,B) and this difference became even more pronounced when focusing specifically on the *S. aureus* to *S. hominis* ratio (Figure S4C,D).

### 3.3 Lipid–Microbiome Correlation Networks Indicate Diminished and Microbe‐Focused Lipid–Microbiome Interactions in Lesional AD

**FIGURE 3:**
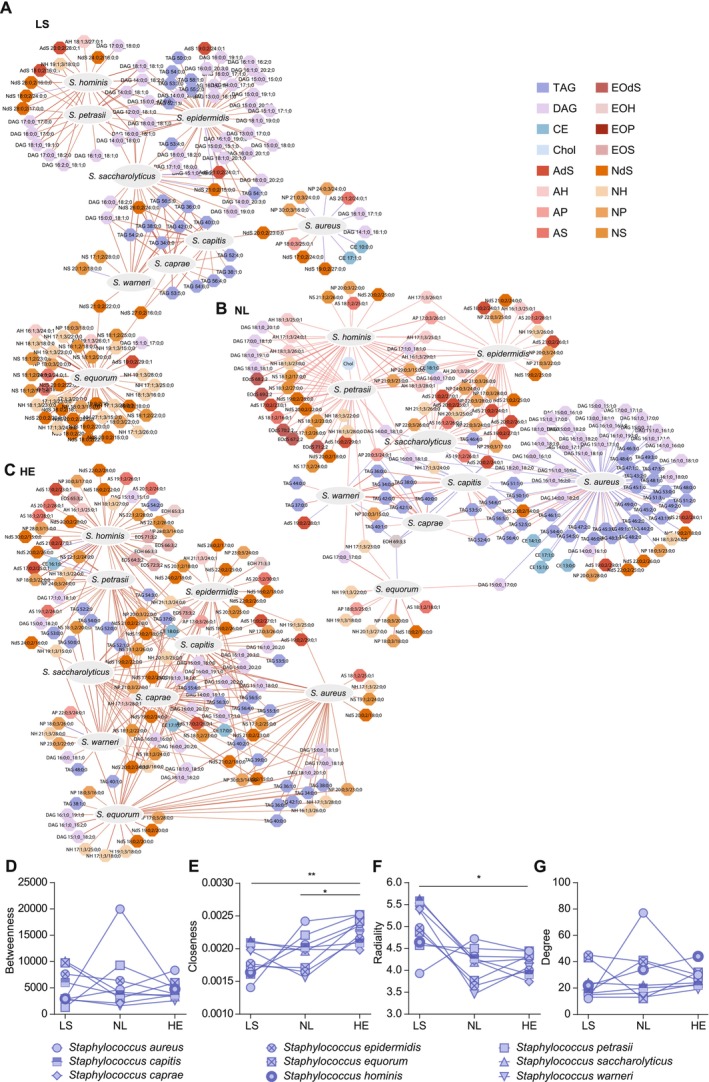
Correlation of epidermal lipids and microbes and their interconnection in atopic dermatitis (AD). Correlation of abundance between molecular lipids and microbe species shown in separate networks for LS (A), NL (B), HE (C). Positive correlations PCC ≥ 0.5 between microbes and lipids are depicted in red and negative correlations (PCC ≤ −0.5) are depicted in purple. Network architecture was analyzed by common centrality parameters such as radiality, closeness, betweenness and degree in the corresponding condition specific networks (D–G). Statistical comparisons between LS vs. HE and NL vs. HE were performed using the Mann–Whitney *U* test. *p < 0.05, **p < 0.01. Interactive network figures are deposited online for LS (A): https://www.ndexbio.org/index.html#/network/f85a6c3e‐6bea‐11f0‐a218‐005056ae3c32?accesskey=12a73b6f2a81cbcfca47362d5cf740dc6d86411126e8f02a1a9f079288a80375 for NL (B): https://www.ndexbio.org/index.html#/network/2a1ac3e3‐6bf2‐11f0‐a218‐005056ae3c32?accesskey=934593f3048f232edae028d6ac7b5a9a016867e2a52f99eb6eaaff00189aec34 and HE (C): https://www.ndexbio.org/index.html#/network/4c0e7f28‐6be0‐11f0‐a218‐005056ae3c32?accesskey=60b9f9a1d7f5670873b0e0145d7061b4b632041cfd6746f927b532dce462d12c. HE, healthy matched control; LS, lesional AD; NL, non‐lesional AD; PCC, Pearson's correlation coefficient.

As *Staphylococcus* species dominated differences between LS, NL, and HE, a correlation network was built around these species (Figure 3A–C, Figure S5, Table 2) and centrality measures—betweenness, closeness, radiality, and degree (Figure 3D–G) to capture the different aspects of network topology. While in NL and HE the correlation network was rather dense (diameter = 6 for both NL and NL, shortest path = 3.52 for HE and 3.43 for NL, average betweenness = 390 in HE and 441.2 in NL) with a variety of lipids correlating with multiple microbes, the LS network was rather sparse (diameter = 8, shortest path = 4.58 and average betweenness = 534.6) and most of the microbes showed exclusive correlations with lipid species forming less connected modules (Figure 3 and Table 2). While closeness across species demonstrated a gradual increase from LS to NL and HE (*p* value = 0.007 for LS vs. HE and *p* value = 0.02 for NL vs. HE), radiality decreased across this gradient (*p* value = 0.01 for LS vs. HE) (Figure 3E,F), suggesting that the network became less modular and more interconnected from LS to HE. This trend is exemplarily described for *S. aureus* representing proinflammatory species and *S. hominis* , representing protective species. In HE, *S. aureus* exhibited numerous correlations with both ceramides (45%) and glycerolipids (50%), while in NL, correlations of *S. aureus* were primarily reduced to mostly negative correlations with glycerolipids (~80%) that in turn showed fewer correlations to other *Staphylococci* than in HE. Accordingly, *S. hominis* revealed a marked reduction in the number and diversity of lipid associations in LS, with interaction diversity increasing in NL and HE. Notably, most lipid associations were condition‐specific (Figure S6). In summary, the number of lipids correlating with both *S. aureus* and other *Staphylococcus* species was drastically reduced in LS, indicating the formation of microbe‐centric modules.

**TABLE 2:** Characteristics of microbe‐lipid correlation networks in AD.

	LS	NL	HE	*p* (ANOVA)
Number of nodes	141	182	156	NA
Number of edges	207	275	250	NA
Diameter	8	6	6	NA
Average distance	4.8	3.43	3.5	NA
Max degree	45	77	44	NA
Average degree	2.96	3.1	3.2	0.06
Average radiality	4.18	3.56	3.47	0.0001
Average centroid	−115.028	−158.8	−122.6	NA
Average closeness	0.0014	0.0016	0.0018	0.001
Average betweenness	534.6	441.2	390	0.007

### 3.4 *Staphylococcus hominis* Abundance Correlates With Distinct Lipids in Reconstructed Human Epidermis

**FIGURE 4:**
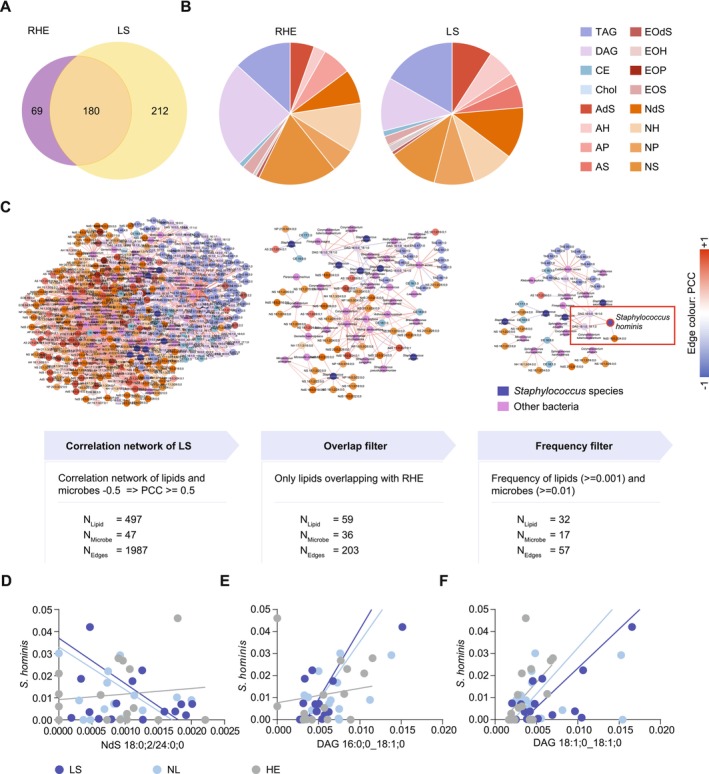
Comparison of lipid profiles between AD and RHE. Overlap of lipids between RHE (*n* = 9) and lipids from the patients' LS skin samples (*n* = 16) (A). Composition of lipid (sub)classes in both RHE and AD (B). The lipid–microbe correlation network in LS was reduced by including only lipids present in both RHE and in vivo skin samples, with lipid and microbe frequencies > 0.001 and ≥ 0.01, respectively. Positive correlations (PCC ≥ 0.5) are shown in red, while negative correlations (PCC ≤ −0.5) are shown in purple. *S. hominis* is encircled in red (C). Positive and negative correlations of *S. hominis* with its three correlating lipid partners NdS 18:0;2/24:0;0 (D), DAG 16:0;0_18:1;0 (E) and DAG 18:1;0_18:1;0 (F) in the filtered network from C. AD, atopic dermatitis; HE, healthy; LS, lesional; NL, non‐lesional; PCC, Pearson's correlation coefficient; RHE, reconstructed human epidermis.

To further study the microbial composition and its interaction with lipid composition in RHE in vitro, lipids from tape‐stripped reconstructed human epidermis cultures (RHE) mimicking AD were compared to those from donors of the study cohort (LS) (Figure 4A,B). 249 lipids were identified from samples of RHE, among which 180 (45.9%) overlapped with 392 lipids from skin samples of the LS patient cohort (Figure 4A). Also, in terms of lipid composition, RHE and LS share a similar distribution of ceramide lipids and glycerol esters (Figure 4B). Next, we aimed at reducing the correlation network between microbes and lipids in LS stepwise by only including lipids that were both present in RHE and samples of the study patients and by removing lipids and microbes whose frequencies in in vivo samples were < 0.001 and < 0.01, respectively (Figure 4C). Within the reduced network containing 32 lipids and 17 microbes, *S. hominis* stood out as a promising candidate for further in vitro analysis as it showed a negative correlation with one NdS species (NdS 18:0;2/24:0;0; *r* = −0.56, *p* = 0.005) (Figure 4D) but was concomitantly positively correlated with two DAGs (DAG 18:1;0_18:1;0 and DAG 16:0;0_18:1;0; *r* = 0.9 and 0.8, respectively, *p* = 0.005 and *p* = 0.005) (Figure 4E,F). As NdS 18:0;2 lipids were found to be more abundant in LS (Figure S3J) as compared to NL and HE, we hypothesized that *S. hominis* might play a beneficial role for the skin barrier by reducing NdS 18:0;2/24;0;0 lipids in the epidermis.

**FIGURE 5:**
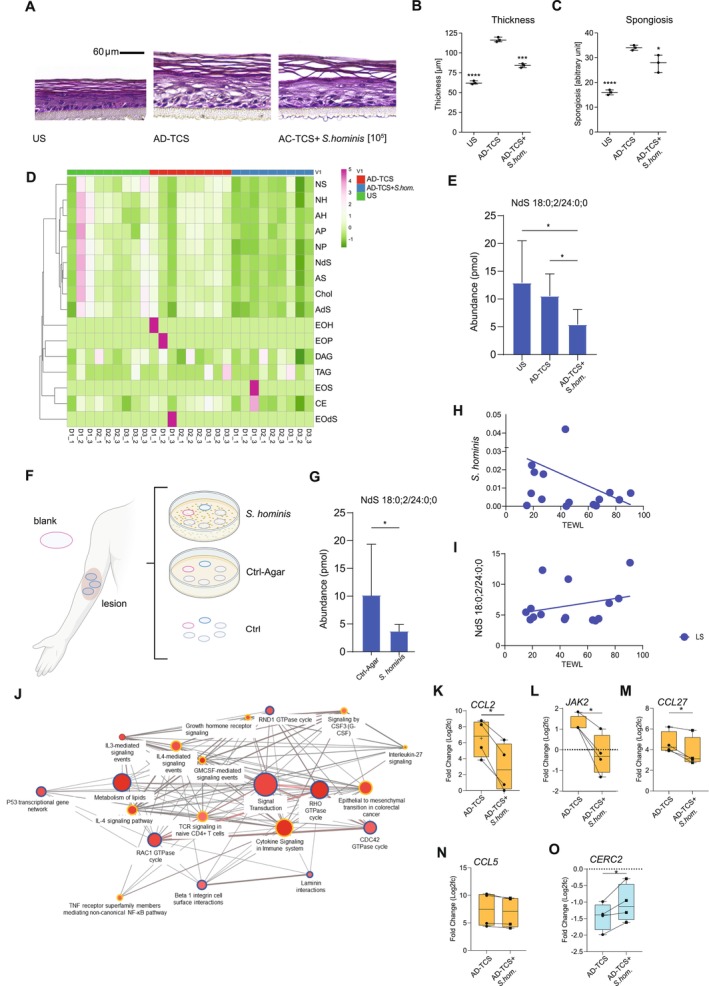
In vitro validation of the interplay between bacteria, lipids and skin barrier. Representative H&E staining of RHE models (*n* = 3) 72 h after AD‐TCS stimulation and bacterial inoculation with *S. hominis* (10^5^) during 24 h of stimulation (A). Scale bar indicates 60 μm. Quantification of the epidermal thickness (w/o stratum corneum) (B) and spongiosis (C) of the RHE models from (A) (*n* = 3). Clustering of lipid classes in RHE models (*n* = 3 and 3 technical replicates), obtained via non‐invasive tape‐stripping and quantified by shotgun mass spectrometry across the conditions US, stimulated with AD‐TCS, and AD‐TCS + *S. hom*. (D). Abundance of NdS 18:0;2/24:0;0 in RHE models from (A) retrieved by non‐invasive tape‐stripping and quantification by shot‐gun mass spectrometry (E). Tape strips collected from lesional skin areas of five atopic dermatitis patients (*n* = 5) alongside corresponding blank controls. Tapes were incubated for short‐term exposure under three conditions: On LB agar plates colonized with *S. hominis* (top), on sterile control agar plates (Ctrl‐Agar, middle), or without contact to agar (Ctrl, bottom) (F). Abundance of NdS 18:0;2/24:0;0 after incubation of tapes on LB agar plates with and without *S. hominis* (G). Pathway networks derived from genes modulated by *S. hominis* in the context of TCS supernatant. Correlation of *S. hominis* (H) and NdS 18:0;2/24:0;0 (I) with trans epidermal water loss (TEWL) in vivo. Pathways enriched in rescued genes (=genes up‐ or downregulated by TCS whose expression was reversed by *S. hominis* co‐treatment) (J). Orange circles: Genes upregulated by TCS. Blue circles: Genes downregulated by TCS. Circle size reflects pathway size, color pathway significance (dark red higher significance, light red lower significance); edge thickness indicates gene overlap between pathways. Boxplots show RNA‐seq fold changes (log2FC) for representative genes AD‐TCS‐stimulated RHE with or without *S. hominis* (K–O). Paired samples from *n* = 4 independent RHE donors. Statistical significance determined by paired two‐tailed *t*‐test: *p < 0.05, ***p < 0.001, ****p < 0.0001. AD, atopic dermatitis; *S. hom*, *Staphylococcus hominis* ; TCS, T cell supernatant; TEWL, trans epidermal water loss; US, unstimulated.

To support this hypothesis, RHE was stimulated with lesional T cell supernatant (TCS) derived from punch biopsies of AD patients (*n* = 2) in the presence or absence of *S. hominis* at a concentration of 10^5^ absolute number of bacteria (Figure 5A). Inoculation of *S. hominis* led to normalization of the AD‐TCS induced skin architecture (Figure 5A), quantified as a reduction of both epidermal thickness (*p* < 0.0001) and spongiosis (*p* = 0.0349) (Figure 5B,C). Moreover, inoculation of *S. hominis* led to a global change in lipid composition (Figure 5D): Hierarchical clustering revealed treatment‐specific patterns, with notable downregulation of alpha‐hydroxyceramides and non‐hydroxy ceramides in the TCS plus *S. hominis* condition (*n* = 3 and 3 technical replicates). Specifically, we validated a progressive decrease in the abundance of the non‐hydroxy ceramide species NdS 18:0;2/24;0;0 from TCS to TCS plus *S. hominis* (Figure 5E) in RHE. To test whether *S. hominis* would directly influence skin lipids, tapes pre‐exposed to lesional skin were placed on LB‐agar plates with *S. hominis* colonies for a short incubation period (Figure 5F, Figure S7). This exposure led to a significant reduction in the abundance of NdS 18:0;2/24:0;0 compared to tapes placed on control agar (Figure 5G). In line with the mutually antagonistic effect of *S. hominis* and NdS 18:0;2/24;0;0 in vitro, *S. hominis* was shown to correlate negatively with transepidermal water loss (TEWL), a measure that increases in proportion to the level of epidermal damage (*r* = −0.29, Figure 5H) in vivo. Accordingly, NdS 18:0;2/24:0;0 correlated positively with TEWL (*r* = 0.27) in vivo (Figure 5I). To assess how *S. hominis* modulates TCS‐induced transcriptional changes, we performed RNA sequencing on RHE models stimulated with TCS alone or in combination with *S. hominis* and calculated a rescue score for each gene. Genes were classified as rescued if *S. hominis* reversed their TCS‐induced expression towards the unstimulated condition. Non‐rescued genes were defined as those further enhanced by *S. hominis* co‐culture. Pathway enrichment analysis revealed that rescued genes related to cytokine signaling such as IL4‐mediated signaling events and lipid metabolism (Figure 5J), while non‐rescued genes mapped to TCR signaling (*IRAK2, TNF*), Rho GTPase cycles, and cell cycle processes (*CDC14A, AURKA*) (Figure S8). Network topology highlighted shared and unique pathway nodes, with greater overlap among immune and signaling modules. Representative Th2 immunity related genes such as *CCL2* [17], *JAK2* [18], *CCL27* [19], *CCL5* [20], as well as *CERS2* [21], an enzyme preferentially synthesizing ceramides with very‐long‐chain fatty acids, were rescued by *S. hominis* treatment (Figure 5K–O), while structural genes such as *COL1A1* were further upregulated. Together, these findings support a dual role for *S. hominis* in modulating inflammation and skin barrier homeostasis.

## 4 Discussion

The epidermal barrier relies on interactions between skin cells, lipids, and commensal bacteria [22, 23]. While individual components have been studied, a combined analysis of the lipidome, microbiome, and in vitro models is lacking. Here, we used quantitative shotgun mass spectrometry to assess skin lipids in lesional and non‐lesional AD and healthy controls. We also analyzed the microbiome and validated an exemplary microbe‐lipid correlation in 3D skin models. Our goal was to explore commensal‐lipid interactions in health and disease and their role in barrier function and skin inflammation.

Unlike conventional spectrometry, we used shotgun mass spectrometry for skin lipid analysis, ensuring higher precision and granularity in lipid subclass identification [6]. While AD is linked to dysbiosis [1, 24], detailed lipid composition remains poorly understood. We found reduced lipid diversity in lesional AD, mirroring microbiome changes, with cholesterol and non‐hydroxyceramides subspecies NS (18:1;2/XX), NdS, and NH dominating. The biological roles of these lipids are unclear, but sphingolipids like ceramide‐1‐phosphate can influence keratinocytes and immune cells [25]. By comparing lesional, non‐lesional, and healthy control samples, we identified disease‐related sphingolipid subclasses, such as NS, NdS, and NH, and potentially protective lipids with a higher expression in non‐lesional and healthy skin, like EOH, EOS, AdS, AH, and AP. These data bear the potential for future therapeutic approaches by supplementing beneficial lipid subspecies instead of use of general moisturizer.

Given this unique dataset of high granularity, we next investigated the correlation between bacteria and lipids in AD and matching controls. Here, we observed clear shifts between healthy and inflamed skin. While there is a multitude of microbe‐lipid correlations in non‐lesional AD and healthy skin, the lesional AD interaction network is restricted to few connections. In addition to the work of Baurecht et al. [3], who showed that unsaturated long‐chain FFAs are correlated with a decreased alpha‐diversity of the microbiome at the antecubital fossa of AD patients, we depicted correlations between single bacteria and lipids. For instance, *S. hominis* positively correlated with ceramides in all healthy skin samples, and *S. aureus* positively correlated to several ceramides and glycolipids in non‐inflamed skin. However, the number of lipids correlating to *S. aureus* decreases tremendously in lesional AD, indicating a potential interrelation between *S. aureus* and reduced lipid diversity. As *S. aureus* produces the lipases sal‐1, sal‐2, sal‐3, and glycerol ester hydrolase, which are specific for short‐ and long‐chain fatty acids [26], it might directly mediate hydrolysis of skin lipids. This hypothesis is supported by the work of Li et al. [27], who reported significantly lower levels of long‐chain ceramides in *S. aureus* positive AD lesions than *in S. aureus* negative lesions. Another possible explanation for the sparse correlation network in lesional AD is that *S. aureus* uptakes, digests, or scavenges skin lipids as a supplement for limited cell components. In fact, Teoh et al. [28] demonstrated that *S. aureus* acquires host‐derived unsaturated fatty acids to overcome branched‐chain fatty acids deficiency, a component of staphylococcal membrane phospholipids. Of note, this uptake is essential to ensure *S. aureus* survival during skin infection.

Finally, we tested if the lipid–microbe interactions detected by bioinformatical analysis represented the natural situation. Therefore, we collected lipid samples from in vitro cultivated RHE, which were exposed to the supernatant of T cells derived from AD skin biopsies. As these models are solely built of keratinocytes, they miss sebaceous units. Therefore, it is inevitable to focus on lipids that are present in both human samples and in in vitro models. We found that almost 80% of the lipids produced by three‐dimensional skin models under Th2 conditions overlap with those detected in human lesional AD samples. Interestingly, there was a strong negative correlation between *S. hominis* , a skin commensal we detected in non‐lesional AD, and a subclass of non‐hydroxyceramide: NdS 18:0;2/24:0;0. When applying *S. hominis* to the skin models, we observed a reduction of NdS 18:0;2/24:0;0, thus proving that the results of the correlation networks closely resemble the biologic interaction between lipids and microbes. To further validate these interactions, we performed RNA sequencing on TCS‐stimulated RHE models with and without *S. hominis* . A subset of TCS‐induced gene expression changes—particularly those related to Th2 cytokine signaling and lipid metabolism—was reversed upon *S. hominis* co‐treatment, including *CERS2* [21], a gene critical for long‐chain ceramide synthesis. Additionally, short‐term exposure of lesional skin‐derived tape strips to *S. hominis* cultures led to a reduction in NdS 18:0;2/24:0;0 levels, supporting a direct lipid–microbe interaction. These findings provide mechanistic support for the regulatory role of *S. hominis* in lipid modulation and barrier stabilization beyond correlative associations.

During the last years, *S. hominis* has attracted increasing attention in the context of AD. *S. hominis* is the second most frequent coagulase‐negative germ isolated from healthy human skin after *S. epidermidis* [15]. Previous studies showed that *S. hominis* negatively correlated with AD severity, while *S. aureus, S. capitis*, and *S. lugdunensis* were highly abundant on lesional AD [29]—a finding which is in line with our observations. Furthermore, *S. hominis* produces six unique autoinducing peptides, which inhibit an important virulence factor (accessory gene regulator‐agr) of *S. aureus* and *S. epidermidis* in vitro and during skin infections in vivo [15]. Third, *S. hominis* *A9* is currently being evaluated as a potential topical therapy for AD. First results confirmed safety and indicated beneficial clinical effects, especially in participants, where a direct killing of *S. aureus* by *S. hominis* *A9* was observed [30]. In line with this emerging data about the protective role of *S. hominis* in AD, we demonstrated that *S. hominis* reduced thickness, spongiosis, and inflammatory gene expression in three‐dimensional skin models in vitro. Hence, there is a direct crosstalk between *S. hominis* and keratinocytes leading to anti‐inflammatory effects within a type 2 inflammatory milieu. Similar mechanisms have been proven for other microbes. For instance, lugdunin, a peptide secreted by *S. lugdunensis* , has direct antimicrobial effects against *S. aureus* but also immune‐modulatory effects on human keratinocytes [31]. Another example is *S. epidermidis* , which releases the lipopeptide LP01, thereby amplifying the production of antimicrobial peptides by keratinocytes [32]. Furthermore, *S. epidermidis* ‐specific IFN‐γ or IL‐17A producing CD8+ cells (Tc1 or Tc17 cells) express a characteristic immunomodulatory gene signature (*OX40L, RANKL, Retinoid X receptor alpha, IL10, IL‐27 beta*) and accelerate wound healing [33]. Our data indicate that *S. hominis* might also mediate direct anti‐inflammatory effects on keratinocytes. However, future studies are necessary to investigate whether direct interactions, pathogen‐associated molecular patterns, or other soluble factors mediate immunomodulatory effects.

This study has some limitations. First, the total study population contains only 16 patients. However, as we performed an in‐depth skin analysis comprising microbiome and lipidome analyses at several body sites, the sample size is comparable to other investigations [3, 34, 35]. Second, 16S rRNA does not allow conclusions about the functional state of bacteria. Therefore, we performed in vitro experiments validating the effects of *S. hominis* in RHE models. To address possible confounders (sex, age, climate), we used age‐ and sex‐matched healthy individuals as controls. Nevertheless, we observed differences in skin lipid composition and microbial diversity dependent on the sample site. This issue was addressed by only focusing on lipid–bacteria interactions which were stable among the majority of samples independent of the sample site.

In summary, we provide a detailed analysis of the lipid–microbiome network in AD, identifying specific lipid signatures of healthy and inflamed skin with potential for targeted lipid supplementation or personalized skincare. By leveraging this dataset, we validated key microbe–lipid interactions in vitro, showing that *S. hominis* can reduce inflammation and restore epidermal architecture in a type 2 immunity environment. Our findings offer new insights into host‐microbe–lipid interactions in AD and open avenues for personalized therapeutic interventions.

## Conflicts of Interest

F.L. reports grants and contracts from Sanofi, Bristol Myers Squibb, Almirall, Novartis, received consulting fees from Amgen, Almirall, Abbvie, Novartis, Leo Pharma, Lilly, UCB, Apogee, J&J, Bristol Myers Squibb, Sanofi, Regeneron, Hexal, Galderma and reports travel grants from UCB, Lilly, J&J. S.W. received honorarium from Sanofi/Regeneron, LEO Pharma, Novartis, Janssen, Almirall, BMS and travel grants from Sanofi/Regeneron, Almirall. T.B. reports grants and contracts from Almirall, Celgene‐BMS; Lilly, Novartis, Sanofi‐Genzyme, Regeneron, Viatris, received consulting fees from AbbVie, Alk‐Abello, Almirall, Boehringer‐Ingelheim, Leo Pharma, Lilly, Novartis, Sanofi‐Genzyme, Viatris, received honorarium from Alk‐Abello, Almirall, Galderma, GSK, Leo Pharma, Lilly, Novartis, Sanofi‐Genzyme, Regeneron and was part of Advisory boards of Alk‐Abello, Almirall, Boehringer‐Ingelheim, Galderma, Leo Pharma, Lilly, Novartis, Sanofi‐Genzyme, Viatris. C.SW reports grants or contracts from Allergopharma and Zeller AG, consulting fees from Leti Pharma and honoraria from Allergopharma and Leti Pharma. K.E. is advisor and/or speaker for Abbvie, Almirall, Apogee, Boehringer Ingelheim, BMS, Galderma, Incyte, Janssen, Moonlake, LEO, Lilly, Novartis, Pfizer, Roche, Sanofi, Sitryx, UCB and holds shares and is co‐founder of Dermagnostix and Dermagnostix R&D. S.E. is co‐founder of Dermagnostix and Dermagnostix R&D. C.T.‐H. reports consulting fees from AstraZeneca, Novartis, Lilly, Sanofi, received honoraria for lectures from Novartis, Lilly, Sanofi, Stallergens, Heuer Dialog GmbH, ALK Abello, Berlin Chemie, BMW Group, Menarini Pharma, Signum PR GmbH, was part of the advisory boards of Sanofi, Novartis, Lilly, Klinge Pharma, is the speaker of the scientific board of CK‐CARE and part of the German advisory council of global change. C.K. is CTO and shareholder of Lipotype GmbH. M.R. reports contracts from Signum PR GmbH, received consulting fees from SebaPharma, received honoraria for lectures from Novartis, Reviderm and was part of the advisory board or Bencard Allergy. N.G.‐S. is advisor and/or speaker for Abbvie, Almirall, Janssen, LEO, Lilly, Novartis, and holds shares and is co‐founder of Dermagnostix and Dermagnostix R&D. Others have no potential conflicts of interest to disclose.

## Supporting information


**Table S1:** Composition of cytokines within AD‐TCS. Concentrations are shown as pg/mL.
**Table S2:** Primer sequences.
**Figure S1:** Classification of lipids.
**Figure S2:** Diversity in lipidome and microbiome.
**Figure S3:** Differentially abundant lipids in AD.
**Figure S4:** Ratio of abundance between inflammatory and protective Staphylococci species.
**Figure S5:** Correlation matrix for molecular lipid and microbiome species.
**Figure S6:** Correlation between lipid molecules and *Staphylococcus hominis* across lesional, non‐lesional and healthy skin.
**Figure S7:** Lipid composition of lesional skin tape samples following co‐incubation with *Staphylococcus hominis* .
**Figure S8:** Pathways enriched in non‐rescued genes and representative gene expression after AD‐TCS stimulation with or without *S. hominis* co‐treatment.

## Data Availability

The data that support the findings of this study are available from the corresponding author upon reasonable request.
