# Supplementary material for: Skin Lipid–Microbe Interplay Links Staphylococcus hominis to Barrier Control in Adult Atopic Dermatitis
Source: Allergy. 2025 Aug 28;81(2):454–67. doi: 10.1111/all.70028 (PMC12862556; doi:10.1111/all.70028)
Supplement: Supplementary file 1 — Table S1: Composition of cytokines within AD‐TCS. Concentrations are shown as pg/mL. Table S2: Primer sequences. Figure S1: Classification of lipids. Figure S2: Diversity in lipidome and microbiome. Figure S3: Differentially abundant lipids in AD. Figure S4: Ratio of abundance between inflammatory and protective Staphylococci species. Figure S5: Correlation matrix for molecular lipid and microbiome species. Figure S6: Correlation between lipid molecules and Staphylococcus hominis across lesional, non‐lesional and healthy skin. Figure S7: Lipid composition of lesional skin tape samples following co‐incubation with Staphylococcus hominis . Figure S8: Pathways enriched in non‐rescued genes and representative gene expression after AD‐TCS stimulation with or without S. hominis co‐treatment. [file ALL-81-454-s001.docx]

**Skin lipid-microbe interplay links *Staphylococcus hominis* to barrier control in adult atopic dermatitis**

**Short title:** Lipibiome in adult atopic dermatitis

**Authors:**

Madhumita Bhattacharyya^1,3,†^, Felix Lauffer^4,5†^, Manja Jargosch^5,6^, Kristina Frey^5^, Mahsa Shahidi Dadras^7^, Theresa Raunegger^5^_,_ Sophia Wasserer^5^_,_ Carsten B. Schmidt-Weber^6^, Tilo Biedermann^5^, Kilian Eyerich^7,8^, Stefanie Eyerich^6^, Claudia Traidl-Hoffmann^1,2,3^, Christian Klose^9^, Matthias Reiger^1,2,3,†^, Natalie Garzorz-Stark^5,7,8,*,†^

**Affiliations:**

^1^Institute of Environmental Medicine Faculty of Medicine and Integrative Health, University Hospital of Augsburg, Stenglinstrasse 2, 86156 Augsburg, Germany

^2^Christine Kühne–Center for Allergy Research and Education (CK-CARE), Davos, Switzerland

^3^Institute of Environmental Medicine, Helmholtz Zentrum München, Augsburg, Germany

^4^Department of Dermatology and Allergy, LMU Hospital Munich, Munich, Germany

^5^Department of Dermatology and Allergy, Technical University of Munich, Munich, Germany

^6^ZAUM – Center of Allergy and Environment, Technical University of Munich and Helmholtz Center Munich, Member of the German Center for Lung Research (DZL), Munich, Germany

^7^Department of Dermatology and Venerology, Medical Center—University of Freiburg, Faculty of Medicine, University of Freiburg, Freiburg, Germany

**^8^**Division of Dermatology and Venereology, Department of Medicine Solna, and Center for molecular medicine, Karolinska Institutet; Stockholm, Sweden

^9^Lipotype GmbH, Dresden, Germany

*To whom correspondence should be addressed:

Prof. Dr. Natalie Garzorz-Stark, PhD, MHBA

Department of Dermatology and Venerology, University of Freiburg

Hauptstraße 7, 79104 Freiburg, Germany

[natalie.garzorz-stark@uniklinik-freiburg.de](mailto:natalie.garzorz-stark@uniklinik-freiburg.de)

^†^ Authors contributed equally

**Keywords**: lipidomics, microbiome, atopic dermatitis, *Staphylococcus hominis*

**Abbreviations:** atopic dermatitis (AD)

**Supplementary Materials and Methods**

*Isolation of lesional T cells from AD skin biopsies and production of supernatant*

Primary human lesional T cells were isolated from freshly taken skin biopsies of AD patients (n=2) by emigration towards an IL-2 gradient followed by expansion with α-CD3/α-CD28 stimulation as described previously^1^. Supernatants of expanded lesional T cells were produced by 3-day stimulation with 0.75 µg/mL α-CD3 (pre-coated, BD Biosciences) and 0.75 µg/mL soluble α-CD28 (BD Biosciences). Concentrations of key cytokines in the supernatant of lesional T cells were determined by ELISA and listed in Supplementary Table S1. A mixture of both two lesional supernatants at equimolar ratio was used for stimulation of RHE.

*Transepidermal water loss*

Transepidermal water loss (TEWL) was measured using the AquaFlux device (Courage+Khazake, Cologne, Germany) following the international guidelines for TEWL assessments^2^. All measurements were performed at 22° C room temperature after 15 minutes of acclimatization.

*Selection of lipids and microbes for in vitro analysis in reconstructed human skin*

To select lipids in AD for further in vitro studies, reconstructed human epidermis (RHE) with primary keratinocytes were stimulated with T cell supernatant to mimic AD (RHE) and tape-stripped for lipid extraction. Lipids distributions were compared between RHE cultures and human donors. A reduced lipid- microbe correlation network was built, including lipids both present in RHE and study patients' skin (≥0.001 frequency). Microbes were included if their frequency in study participants' skin was ≥0.01 and their microbe-lipid correlation was significant (PCC ≥ 0.5 or ≤ -0.5).

*Reconstructed human epidermis (RHE) and Staphylococcus hominis* *inoculation*

As described previously^1^ , RHE was generated by culturing 0.3 × 10⁶ primary human keratinocytes in collagen-coated inserts (Millipore, PIHP01250) within a 6-well plate using keratinocyte medium (DermaLife Basal Medium supplemented with DermaLife K LifeFactor Kit (Lifeline Cell Technology, LL-0007)) with 1.5 mM CaCl₂. After two days, an airlift was performed, and the medium was supplemented with 50 µg/ml vitamin C (SIGMA, A5960-25G). The medium was replaced every two days.

After 12 days, RHE was stimulated with 1:10 diluted T cell supernatant (TCS) from lesional AD skin for 24 h (RNA) or 72 h (lipids/histology). Twenty-four hours before the end of stimulation, models were inoculated with 10⁵ or 10⁶ Staphylococcus *hominis* in PBS, followed by aspiration after 1 hour.

At the end of stimulation, RHE samples were:

- Collected in RLT-Plus buffer (RNA isolation)
- Tape-stripped using D101-Squame discs (lipid sampling)
- Fixed in 4% formaldehyde (histology)

*Histology and H&E staining*

Fixated RHE were divided into two pieces and embedded in paraffin. 5 µm sections were cut and dewaxed at 65 °C for 20 min. After rehydration, sections were stained with hematoxylin and eosin using standard methods. High resolution images (20x) were obtained with Evos microscope. *Quantification:* Epidermal thickness and spongiosis was quantified using ImageJ measurement tool.

*RNA analysis*

RNA from keratinocyte skin equivalents was isolated using RNeasy Mini Plus Kit (Qiagen) according to manufacturer’s protocol. mRNA was transcribed into cDNA with Applied Biosytems High Capacity cDNA Reverse Transcription Kit (Thermo Fisher Scientific). Gene expression was measured on an Applied Biosystems ViiA7 Real-Time PCR system (Thermo Fisher Scientific) using Fast Start Universal SYBRGreen Master Rox (Roche). Primers were ordered from Metabion (http://www.metabion.com) and are listed in supplementary Table S2. Data are normalized to housekeeper gene *18S* (Δct) and shown as relative gene expression to stimulation control AD-TCS using 2^-(ΔΔct)^ method.

**Supplementary Tables**

Table S1: Composition of cytokines within AD-TCS. Concentrations are shown as pg/ml.

| pg/ml | IL-4 | IL-13 | IL-22 | IL-6 | IL-17A | TNF-α | IFN-γ |
| --- | --- | --- | --- | --- | --- | --- | --- |
| AD1 | 4645 | 73869 | 8933 | 0 | 0 | 2720 | 0 |
| AD2 | 3733 | 45729 | 1734 | 3 | 0 | 3277 | 0 |

Table S2: Primer sequences.

| Gene | Sequence (5’-3’) | |
| --- | --- | --- |
| *18S* | fw | GTAACCCGTTGAACCCCATT |
|  | rv | CCATCCAATCGGTAGTAGCG |
| *CCL2* | fw | TCCCAAAGAAGCTGTGATCTTCA |
|  | rv | TTTGCTTGTCCAGGTGGTCC |
| *COL1A1* | fw | CTGGCCTCCCTGGAATGAAG |
|  | rv | GGCAGCACCAGTAGCACC |
| *DSC1* | fw | GCTTGGCGAAGAATCCATTAGAG |
|  | rv | TCCCTCTTCTTCCTGCCGAT |
| *IL36G* | fw | AGGAAGGGCCGTCTATCAATC |
|  | rv | CACTGTCACTTCGTGGAACTG |
| *KRT10* | fw | GTCCCAACTGGCCTTGAAAC |
|  | rv | GGGCCTGAATCTGTGAGAGC |
| *KRT16* | fw | TATTCTTCCCGCGAGGTCTTCAC |
|  | rv | TGGATGAGCTCTGCTCCTTGA |
| *MMP2* | fw | TGCATCCAGACTTCCTCAGGC |
|  | rv | CCTGGCAATCCCTTTGTATGTTT |
| *S100A7* | fw | TCCAAACACACACATCTCACT |
|  | rv | TCTTGTCATCACGTCTGGTGT |
| *S100A8* | fw | TTTCAGGTGGGGCAAGTCC |
|  | rv | CGTCTGCACCCTTTTTCCTGATA |
| *SERPINB3* | fw | GGCAGCAATACCACATTGGTTC |
|  | rv | GGACTTGTATGTATTCTTGTTTGGC |

**Supplementary Figure and Figure Legends**

**
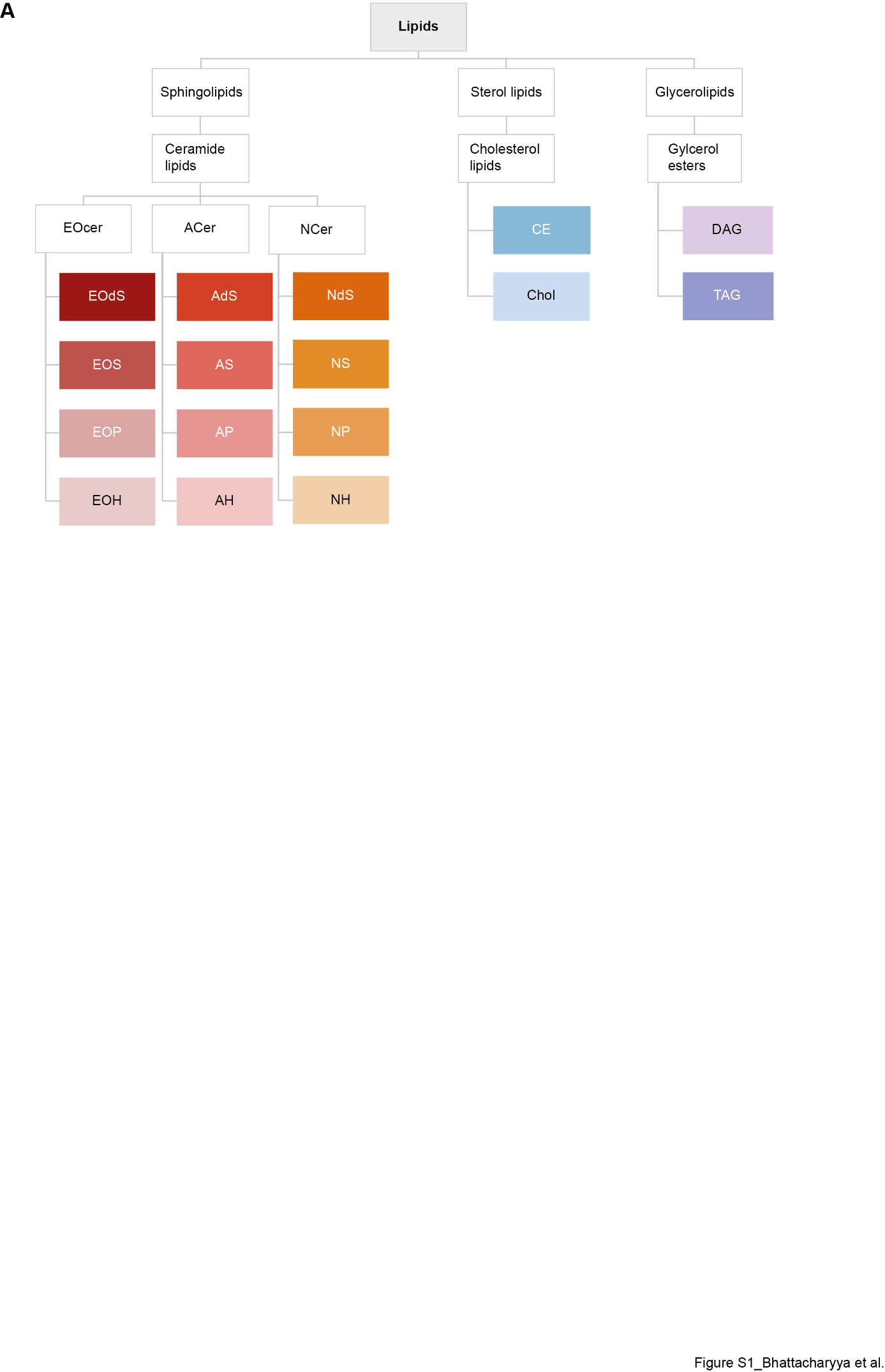
**

**Figure S1: Classification of lipids.** Lipids are shown in categories (Sphingolipids, sterol lipids, glycerlipids), groups (ceramide lipids, cholesterol lipids, glycerol esters), classes (Chol= Cholesterol; CE=Cholesteryl Ester, DAG= Diacylglycerol, TAG=Triacylglyerol, EOcer=Omega-hydrody ceramides, ACer= Alpha-hydroxyceramides, Ncer= Non-hydroxy ceramides) and subclasses (AdS= Alphahydroxy-dehydrosphingosine, AH= Alphahydroxy-6-hydroxysphingosine, AP= Alphahydroxy-phytosphingosine,  AS= Alphahydroxy-sphingosine, EOdS= Omegahydroxy-dehydrosphingosine , EOH= Omegahydroxy-6-hydroxy-sphingosine, EOP= Omegahydroxy-phytosphingosine, EOS= Omegahydroxy-sphingosine,  NdS= Non-hydroxy-dehydrosphingosine , NH= Non-hydroxy-6-hydroxysphingosine, NP= Non-hydroxy-phytosphingosine, NS= Non-hydroxy-sphingosine)

**
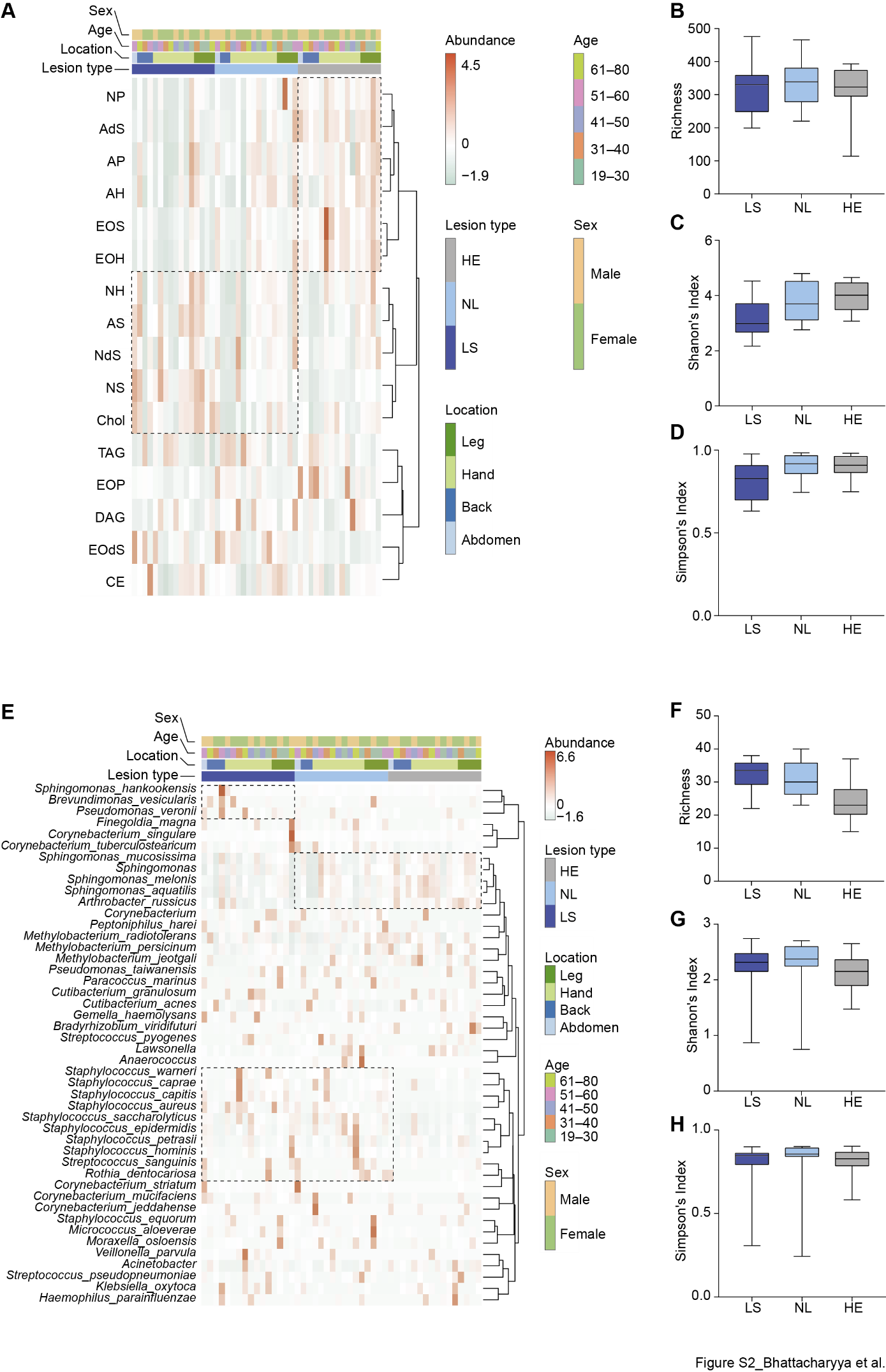
**

**Figure S2: Diversity in Lipidome and Microbiome**. Hierarchical clustering of lipid (A) and microbes (E) on class (lipids) and species level (microbes) within LS, NL and HE. Healthy control samples were listed in the same order as corresponding matching lesional/non-lesional samples. Alpha diversity indices Richness, Shannon’s Index and Simpson’s Index are presented for both lipidome (B-D) and microbiome (F-H). AD = atopic dermatitis, HE = healthy, LS = lesional, NL = non-lesional

**
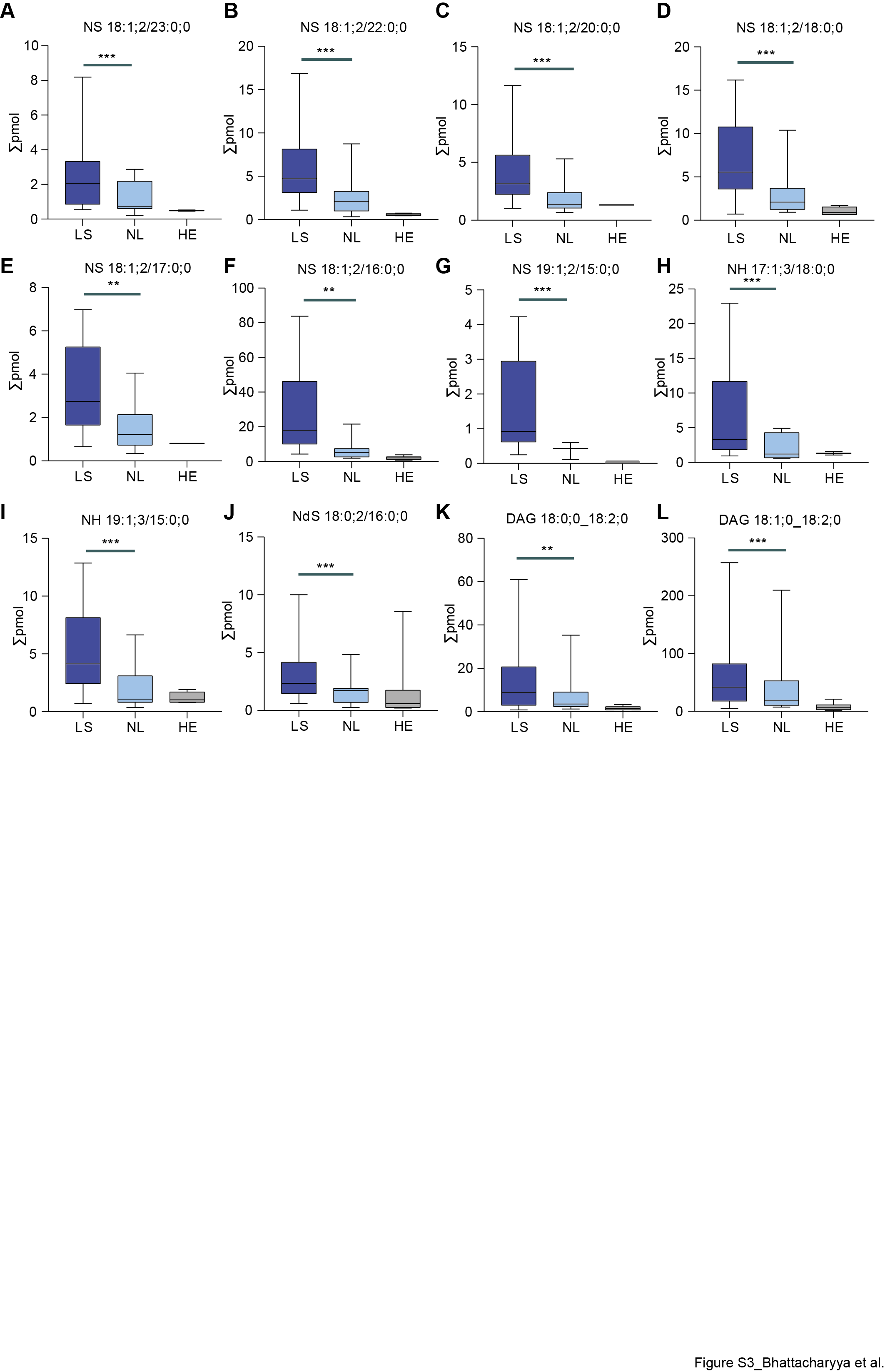
**

**Figure S3:** **Differentially abundant lipids in AD.** Abundance of top 12 differentially abundant lipid species depicted for each group. AD = atopic dermatitis, HE = healthy, LS = lesional, NL = non-lesional


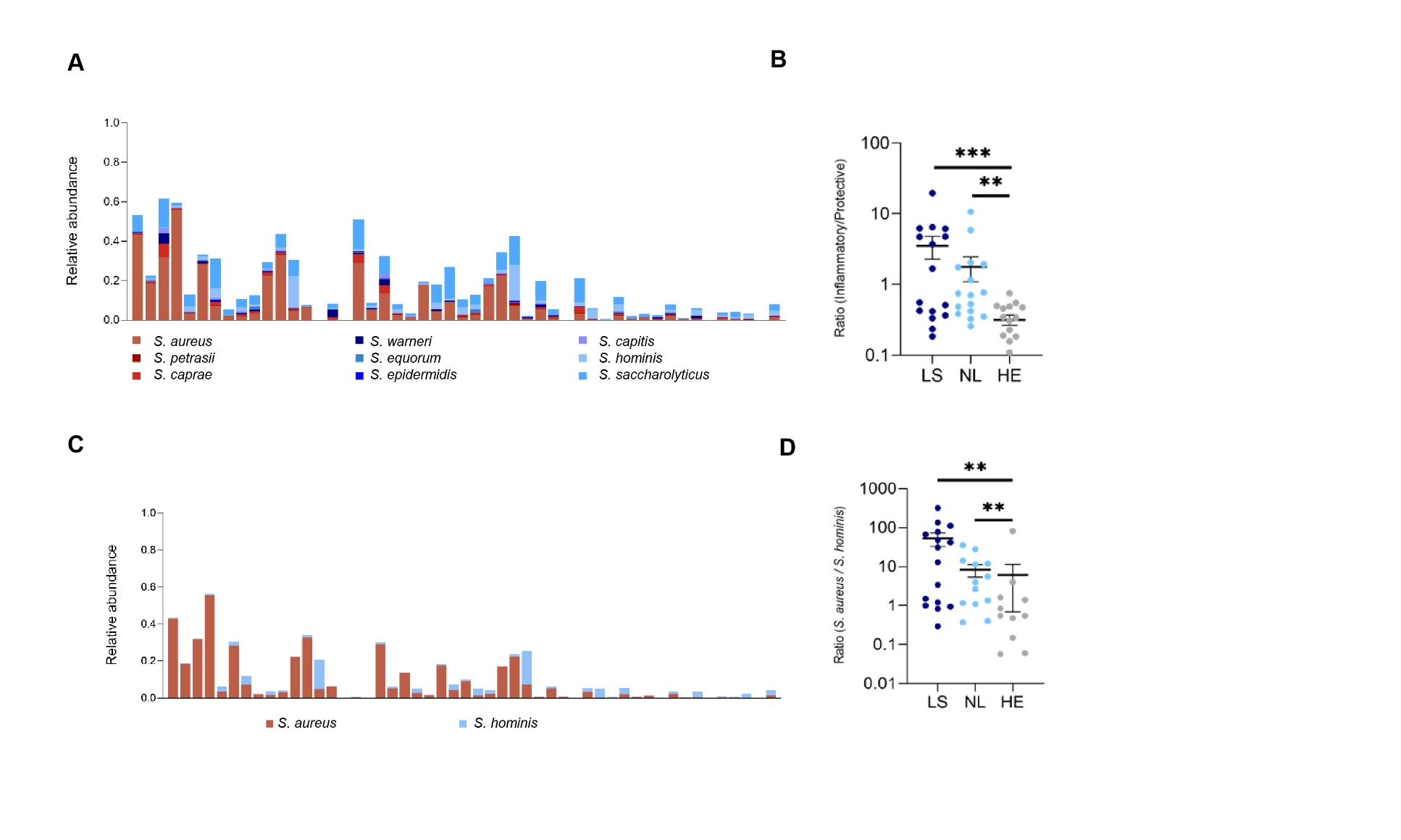


**Figure S4: Ratio of abundance between inflammatory and protective Staphylococci species.** Relative abundance and ratios of *Staphylococcus* species in lesional, non-lesional, and healthy skin (A). Stacked bar plots of the relative abundance of selected *Staphylococcus* species grouped by previously described inflammatory (*S. aureus*, *S. caprae*, *S. petrasii*, shades of red) and commensal/protective species (*S. hominis*, *S. epidermidis*, *S. warneri*, *S. saccharolyticus*, *S. capitis*, *S. equorum*; shades of blue). Ratio of the combined relative abundance of inflammatory to commensal species across LS, NL, and HE skin; higher ratios in LS indicate a shift toward proinflammatory *Staphylococcus* communities (B). Focused comparison of *S. aureus* and *S. hominis* relative abundances across samples (C). Ratio of *S. aureus* to *S. hominis* abundance (D). HE = healthy, LS = lesional AD, NL = non-lesional AD. p < 0.01, *p < 0.001.


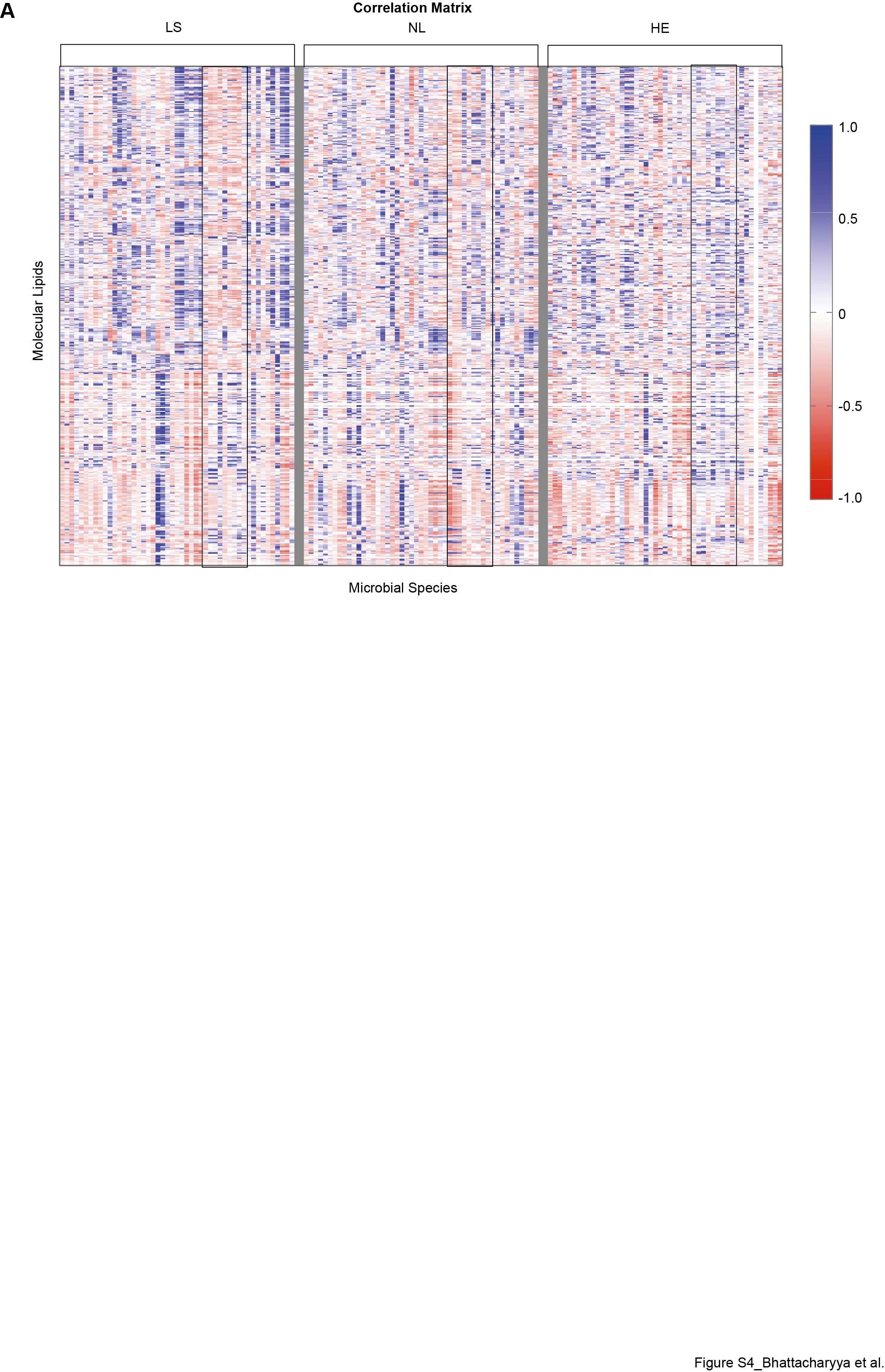
**Figure S5: Correlation matrix for molecular lipid and microbiome species.** All-to-all correlation is calculated among molecular lipids and microbe species. The correlation matrix reveals disease specific patterns along with the dynamic changes from healthy to lesional tissue. Black boxes mark the correlations which were selected for the creation of the network in figure 3. HE = healthy, LS = lesional AD, NL = non-lesional AD


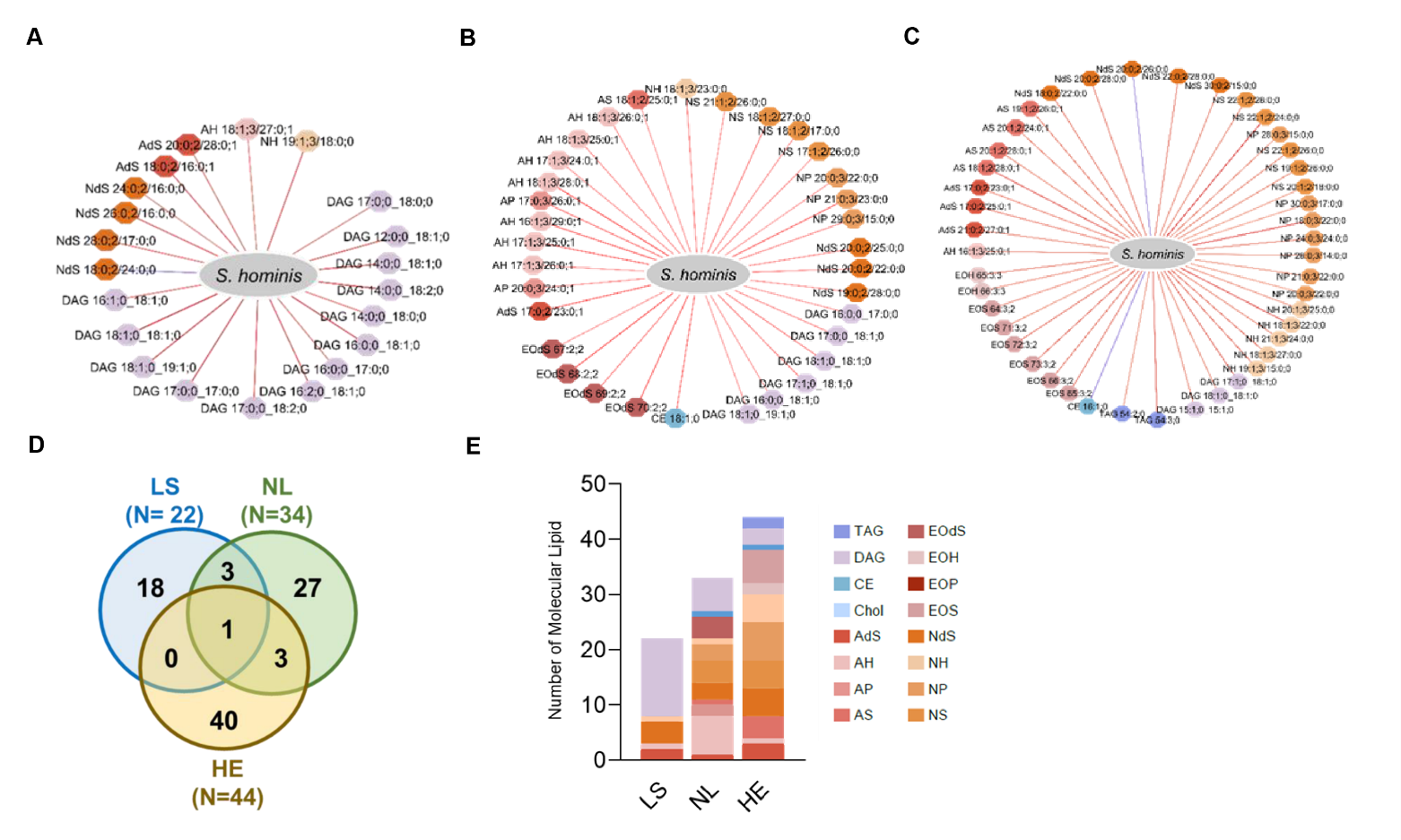
**Figure S6: Correlation between lipid molecules and *Staphylococcus hominis* across lesional, non-lesional and healthy skin.** Pearson correlation networks between *S. hominis* abundance and individual lipid molecules in LS, NL, and HE skin, respectively (A–C). Red (Blue) edges indicate positive (negative) correlations. (D) Venn diagram illustrating the number of lipid species correlated with S. *hominis* in LS, NL, and HE. Each skin condition shows a distinct set of lipid associations, with minimal overlap across states. (E) Stacked column plot showing the distribution of lipid subclasses among the *S. hominis*-associated lipids for each condition. Lipid class abbreviations s. Figure S1. HE = healthy, LS = lesional AD, NL = non-lesional AD.
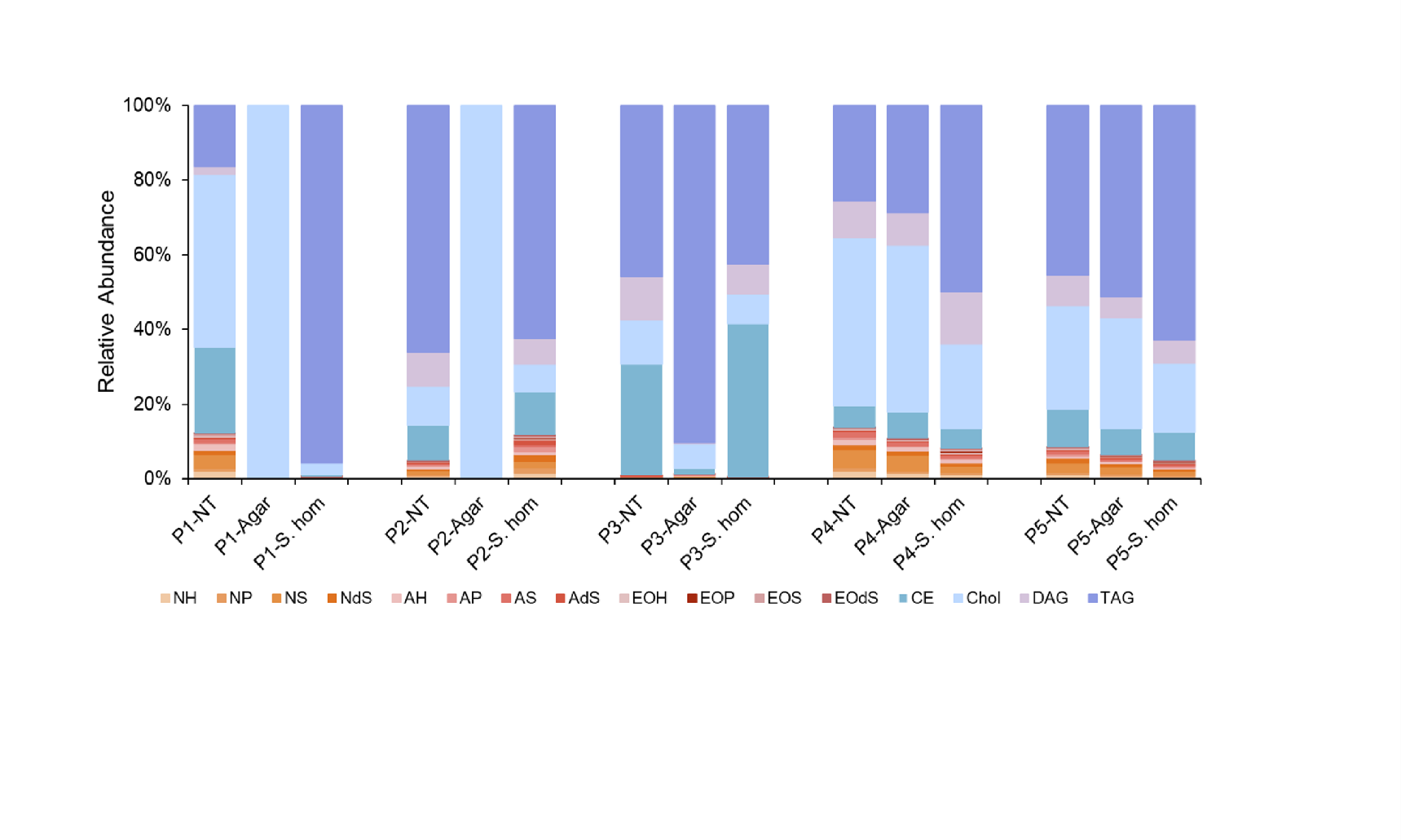


**Figure S7: Lipid composition of lesional skin tape samples following co-incubation with *Staphylococcus hominis*.** Tape strips obtained from LS of five patients (P1–P5) were placed on either (i) empty agar (Agar) or (ii) agar plates cultured with *S. hominis* (*S. hom*), and incubated for 6 h. Untreated tapes (NT, blanks) served as controls. Stacked bar graphs display the relative abundance of lipid classes for each patient and condition. Samples from P1 and P2 showed near-complete lipid loss upon agar exposure, likely due to adherence to the agar surface, and were excluded from further statistical analysis. Lipid class abbreviations s. Figure S1.

**
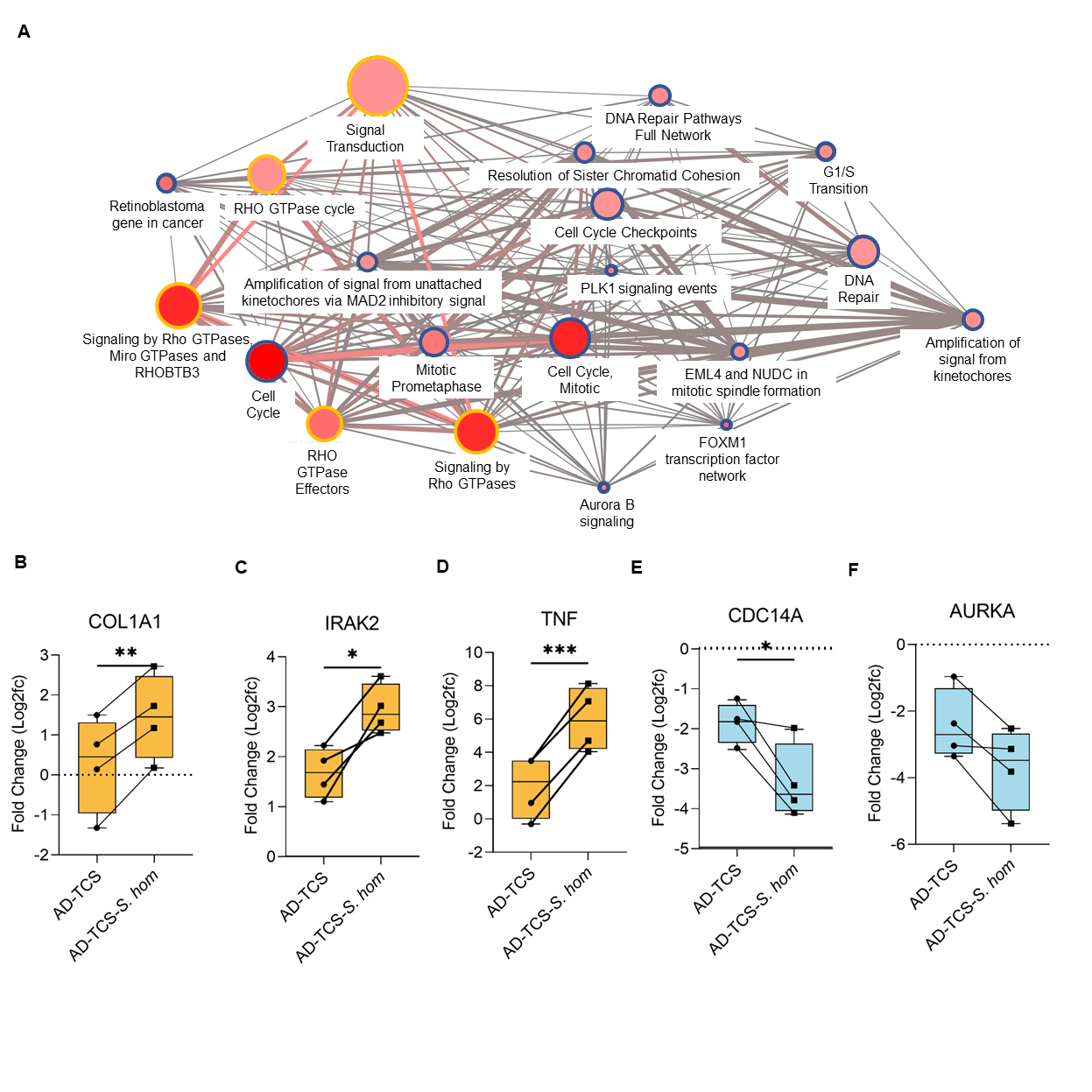
**

**Figure S8: Pathways enriched in non-rescued genes and representative gene expression after AD-TCS stimulation with or without *S. hominis* co-treatment.** Pathway network derived from non-rescued genes—genes significantly up- or downregulated by AD-TCS whose expression was further enhanced by *S. hominis* co-treatment (A). Orange circles indicate pathways enriched in genes upregulated by TCS; blue circles indicate pathways enriched in genes downregulated by TCS. Circle size reflects the number of genes per pathway; color intensity denotes significance (dark red = high significance). Edge thickness represents gene overlap between pathways. Boxplots show RNA-seq fold changes (log_2_FC) for representative genes COL1A1, IRAK2, TNF, CDC14A, and AURKA in AD-TCS-stimulated RHE with or without *S. hominis* (B–F). Paired samples from n = 4 independent RHE donors. Statistical significance determined by paired two-tailed t-test: *p < 0.05, **p < 0.01, ***p < 0.001.

**References**

1. Lauffer F, Jargosch M, Krause L, et al. Type I immune response induces keratinocyte necroptosis and is associated with interface dermatitis. J Invest Dermatol. Mar 8 2018;doi:10.1016/j.jid.2018.02.034

2. Pinnagoda J, Tupker RA, Agner T, Serup J. Guidelines for transepidermal water loss (TEWL) measurement. A report from the Standardization Group of the European Society of Contact Dermatitis. Contact Dermatitis. Mar 1990;22(3):164-78. doi:10.1111/j.1600-0536.1990.tb01553.x
